# Trauma-informed peacebuilding: a systematic mapping review of training programs

**DOI:** 10.1186/s13031-026-00773-6

**Published:** 2026-02-19

**Authors:** Lars Dumke, Susanne Heumann-Schoop, Emily Brandenburg, Malte Behrendt, Ingo Schäfer

**Affiliations:** Department of Psychiatry and Psychotherapy, University Medical Center Hamburg-Eppendorf, Hamburg, Germany

**Keywords:** Peacebuilding, Mental health and psychosocial support (MHPSS), War, Conflict, Trauma, Post-conflict recovery, Humanitarian settings, Trauma-informed approaches, Trauma sensitive

## Abstract

**Background:**

The psychosocial consequences of conflict and trauma exposure place a significant burden on individuals, communities, and on the social fabric essential for rebuilding peace. There is growing recognition of the need to integrate trauma-informed approaches into peacebuilding efforts. However, little is known about how practitioners are being equipped for this work, what training programs exist, and what core components they include.

**Methods:**

This systematic mapping review aims to systematically map and characterize existing training programs on trauma-informed peacebuilding, specifically examining their core components and implementation modalities. An initial systematic search of bibliographic databases (Web of Science, Scopus, PubMed) yielded no relevant results. Therefore, a systematic web search of non-bibliographic material was conducted in line with Cochrane’s guidelines. A total of 2,400 results were screened, and 21 relevant training initiatives were included.

**Results:**

The review identified 15 facilitated and six non-facilitated programs. Training providers were primarily non-governmental organizations based in high-income Western countries, with half of all programs originating in the United States. Thematic analysis of training contents revealed seven core components: (1) defining and conceptualizing trauma, (2) realizing the impact of trauma on peace and peacebuilding efforts, (3) self-care and resilience strategies for peace practitioners, (4) psychosocial support skills to address trauma and foster resilience in communities, (5) integrating trauma-informed practices into programs and organizations, (6) ethical considerations, and (7) cultural competence and contextual awareness.

**Discussion:**

Despite increasing demand, training efforts in trauma-informed peacebuilding remain fragmented, unevenly distributed, and unevaluated. This review highlights the need for more accessible, contextually relevant, and evidence-informed training initiatives. Recommendations are provided to guide future training development and inform policy and practice aimed at strengthening trauma-informed capacities in peacebuilding.

**Supplementary Information:**

The online version contains supplementary material available at 10.1186/s13031-026-00773-6.

Armed conflict can have profound psychological and social consequences. It is estimated that at least one in five individuals in conflict-affected settings experiences a mental health condition such as depression, anxiety, post-traumatic stress disorder (PTSD), bipolar disorder, or schizophrenia [1, 2]. The psychosocial consequences of conflict-related trauma place considerable strain on individuals, families, communities and on the social fabric that is essential for rebuilding trust, relationships, and a peaceful future.

Trauma exposure, i.e., experiencing extremely threatening or horrific events such as bombings, torture, or sexual violence, has been linked to a heightened sense of threat, mistrust, and difficulties with emotional regulation and interpersonal functioning among affected populations [3–5]. Consequences may further include avoidance and social withdrawal, aggressive or violent behavior, delinquency, or the use of harmful coping strategies, such as substance misuse [6–9]. For example, among youth in eastern DR Congo, severe trauma histories - particularly those involving both experiencing and perpetrating violence during conflict - were associated with higher levels of PTSD symptoms as well as ongoing aggression and offending behaviors, such as stealing, damaging property, and physical assault [8]. In Iraq, trauma exposure among young adults imprisoned for terrorism-related crimes was linked not only to high rates of PTSD and depression, but also aggression and diminished expectations for reintegration into society [9, 10]. It becomes apparent that in addition to the psychological suffering of the individual, conflict can have lasting negative consequences on the social processes, dynamics, networks, institutions, capital and resources of affected communities [11, 12]. This collective trauma can shape the way communities view the world and their relationships with other groups, further eroding social trust, beliefs, norms, and cohesion [12, 13]. When unaddressed, these consequences may become intergenerational, passed down from one generation to the next [13, 14]. Consequently, divisions in society may be perpetuated over time, preventing the (re-) formation of social bonds, and hindering dialogue and reconciliation. Hence, unaddressed trauma has been identified as a factor that can contribute to recurring cycles of violence and pose a significant obstacle to peacebuilding efforts and the healing of societies affected by conflict [8, 15, 16].

In recent years, there has been a growing recognition of the need to build trauma-informed services and integrate mental health and psychosocial support (MHPSS) into peacebuilding interventions [17, 18]. Peacebuilding encompasses a wide range of processes, approaches, and activities aimed at addressing the underlying causes of conflict and transforming relationships, structures, and cultural dimensions to support sustainable peace [19]. It seeks to understand why conflicts emerge, enable individuals and communities to manage them non-violently, and strengthen social trust and cohesion to prevent the recurrence of violence. As relationships are central to peacebuilding, this work typically involves close engagement with trauma-affected individuals and communities. Guidance documents from international agencies such as the United Nations Development Programme (UNDP) and the Inter-Agency Standing Committee (IASC) now provide frameworks and recommendations for operationalizing the integration of MHPSS and peacebuilding [20, 21]. These frameworks emphasize the importance of applying MHPSS principles, knowledge, and tools to peacebuilding initiatives - and vice versa [20, 21].

In peacebuilding contexts, practitioners regularly encounter the profound psychological and social consequences of trauma within the communities they support [15, 22, 23]. In a global IASC survey of 165 peacebuilding professionals working in 29 countries, participants emphasized that a peaceful society cannot exist if trauma remains unaddressed in individuals, families, and communities [20]. Building a foundational understanding of mental health and trauma-informed care is therefore crucial for effective and lasting peacebuilding. Common definitions of trauma-informed approaches emphasize an understanding of the widespread impact of trauma on individuals, communities, and systems, as well as the recognition of potential paths for recovery [24–27]. A trauma-informed approach typically involves recognizing the signs of trauma, integrating trauma awareness into policies and practices, and taking active steps to prevent retraumatization of clients as well as staff [24, 25, 27]. ​​Importantly, becoming trauma-informed is not a matter of applying a fixed set of techniques, but rather requires ongoing reflection, organizational change, and adherence to key principles such as safety, trustworthiness, choice, collaboration, empowerment, and cultural sensitivity [24–26]. When applied to peacebuilding, a trauma-informed approach can help ensure that programs are responsive to the needs of trauma-affected populations, contribute to both individual healing and the restoration of social cohesion, as well as protect the wellbeing of peacebuilding practitioners [15, 22, 23].

Despite growing demand for trauma-informed peacebuilding, there is limited knowledge about how practitioners are currently being equipped for this work. Previously, peacebuilding professionals reported a lack of training and mentorship in key competencies, including knowledge, skills and attitudes relating to mental health and trauma-informed care, as a major challenge in their work [20]. However, to date, no review has mapped how trauma-informed peacebuilding training programs are structured and implemented. It remains unclear what training programs are available, what core components they include, how they are structured and delivered, and to what extent they align with key principles of trauma-informed care. This lack of insight may undermine efforts to effectively build practitioner capacity, and ultimately risks undermining efforts to foster resilience and build sustainable peace.

This review aims to map the global landscape of trauma-informed training programs for peacebuilding practitioners. Moreover, it aims to identify and characterize available programs, including their content, delivery formats, and target audiences, and highlights common themes and gaps across initiatives. By offering recommendations for future training development, the review seeks to inform both practice and policy aimed at strengthening trauma-informed capacities in peacebuilding efforts.

## Methods

### Study design and search rationale

We conducted a systematic mapping review to comprehensively identify, organize, and synthesize available information on trauma-informed peacebuilding training programs, in order to describe the field, highlight gaps, and inform future research and practice [28]. An initial search of bibliographic material in Web of Science, Scopus, and PubMed yielded no relevant results on training programs for trauma-informed practice in peacebuilding settings (see Supplement S1). Consequently, a systematic web search was conducted, as this approach was deemed particularly effective for identifying non-bibliographic data and capturing information in rapidly evolving fields, such as the integration of MHPSS and trauma-informed approaches into peacebuilding programs. Our search strategy adhered to the Cochrane guidelines for web searching in systematic reviews of interventions [29]. Additionally, when applicable, the review followed the Preferred Reporting Items for Systematic Reviews and Meta-Analyses extension for Scoping Reviews (see Supplement S2 for PRISMA-ScR Checklist) [30]. No protocol was pre-registered for this review.

### Eligibility criteria

Training programs or materials were included if they met the following criteria: (1) provided publicly available information (e.g., information on course objectives and contents, manuals), (2) targeted organizations, staff, or individuals engaged in peacebuilding activities, (3) included components focused on trauma-related knowledge or skill development, (4) were implemented after 2010, and (5) were documented in English or German. Search results were excluded if the described training programs or material lacked relevance to peacebuilding (e.g., targeting professionals without any focus on conflict transformation, reconciliation, post-conflict recovery) or did not provide publicly accessible details about their content.

### Systematic search strategy

To identify relevant training programs for trauma-informed peacebuilding, a comprehensive web search was conducted using Google (www.google.com) and the metasearch engine Metacrawler (www.metacrawler.com) in December 2024. The search strategy combined keywords related to trauma, peacebuilding, and training to twelve search strings (Supplement S3). Given the high volume of results and the decreasing relevance beyond the first few pages, only the first 100 results per search string were screened, consistent with the approach used in previous reviews [31]. Potentially relevant search results were accessed and linked pages or documents assessed in detail for eligibility.

### Study selection

**Fig. 1:**
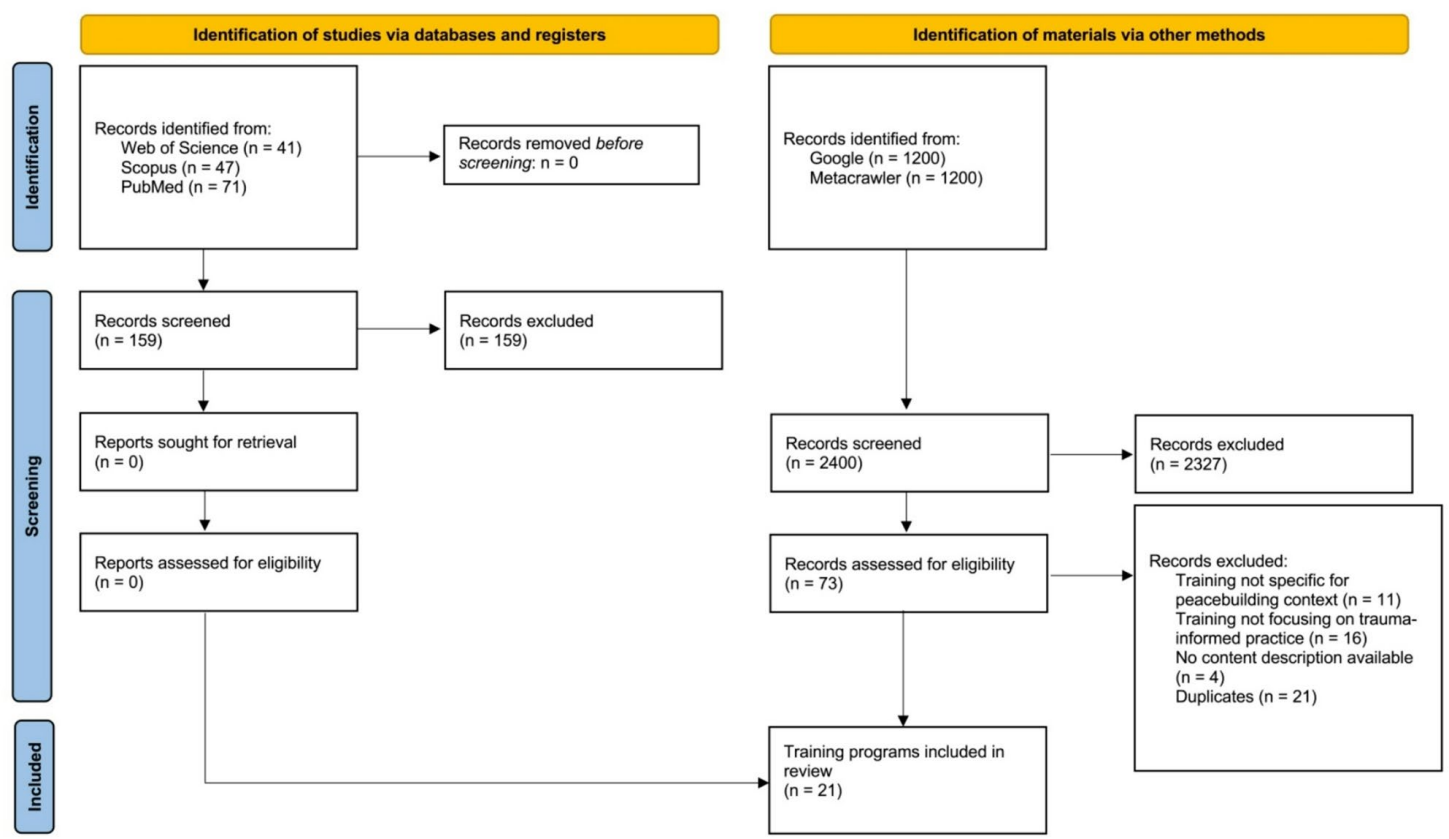
Flow chart of study selection process

A total of 2,400 search results were screened. Of these, 73 were examined in detail for inclusion. The study selection process is presented in Fig. 1. Two reviewers independently screened the results and assessed the eligibility of potentially relevant materials. Prior to formal screening, both reviewers jointly screened an initial subset of 100 search results to calibrate the application of eligibility criteria and discuss areas of ambiguity. The remaining records were then screened independently, with high interrater agreement (> 90%). In addition, the Authority, Accuracy, Coverage, Objectivity, Date, Significance (AACODS) checklist for appraising diverse formats of grey literature was used to appraise the quality and credibility of each identified source [32]. The AACODS appraisal was conducted for transparency purposes and did not inform inclusion decisions. A summary of the quality appraisal is provided in the supplemental material (Supplement S4). Any uncertainties regarding the eligibility and quality of included materials were resolved through discussion among the reviewers.

### Collating, summarizing, and reporting the results

From each identified training program, the following data were extracted with a standardized extraction form: title of training program, name of training provider, type of training provider (e.g., non-governmental organization, international organization, governmental organization), geographical location of training provider, professional background and expertise level of target audience, enrollment prerequisites, training duration, delivery modality (e.g., on-site, online, e-learning, blended), training methods (e.g., case studies, group work, role plays), and program content. The extracted data on training content were examined for recurring and overlapping themes using thematic analysis [33]. The analytic steps included charting all material, familiarization with the data, generation of initial codes, identification of recurring and overlapping content, and the grouping of codes into broader themes. These themes were reviewed, refined, and defined through discussion among the reviewers. A narrative synthesis was then developed to describe the most prominent themes across the identified training programs.

## Results

### Characteristics of included trauma-informed peacebuilding training programs

A total of 21 training initiatives focused on trauma-informed peacebuilding were identified and included in this review, comprising 15 facilitated and six non-facilitated programs. An overview is provided in Table 1. The training programs were offered by a variety of providers, with 13 (62%) offered by non-governmental organizations (NGOs), five (24%) by academic institutions, and three (14%) by government-affiliated or multilateral organizations.

**Table 1:** Overview of training programs included in the review

Ref.	Name of Training Provider	Title of Training Program	Type of Training Provider	Geographical Location of Training Provider	Fees	Target Audience	Enrollment Prerequisites	Training Duration	Delivery Modality	Training methods
**Facilitated training:**										
[37]	Alliance for Middle East Peace (ALLMEP)	Trauma in Peacebuilding	NGO (coalition of 160 organizations)	USA	N/A	Program managers, field staff, and psychosocial support practitioners	Open only to staff from ALLMEP member NGOs	Online component: 4 monthly sessions (session duration not specified) In-person component: Three-day workshop (duration not specified)	Online and in-person	Online sessions, workshops including practical skill-building, WhatsApp groups and access to online resources and tools for ongoing support
[52]	Hartford International University for Religion and Peace	Peacebuilding Skills: Dialogue, Trauma & Restorative Justice	Academic institution	USA	N/A	Students	Restricted to students at Hartford International University	56 h (7 days x 8 h)	In-person	Case studies, role-plays, guest speakers, reading, creating presentations, discussions
[44]	International Association for Human Values (IAHV)	Towards Integrated Peacebuilding	NGO	Switzerland	€580	Individuals and organizations working in peacebuilding, humanitarian, development, security, justice sectors	Not specified	30 h (7 sessions) + 11 h individual practice	Online	Live sessions, individual exercise, group work
[40]	Kurve Wustrov	Stress and Trauma Sensitivity in Peacebuilding	NGO	Germany	from €1000 incl. materials and lodging; reduced fee/travel support possible	Staff of NGOs or initiatives who promote nonviolence, peace, and justice	Application necessary, English proficiency	5 days	In-person	Not specified Post-training-support through email or messenger
[43]	Center for International Peace Operations (ZIF)	Trauma-Sensitivity in Peace Operations	Government affiliated organization	Germany	€400	Civilian experts, police and military personnel who serve in peace missions or other field operations	Application necessary, short pre-course needs assessment, English proficiency	18 h (4 sessions x 4.5 h) + self-paced learning	Online	Live sessions, digital tools and resources, individual learning, group work
[47]	Mediators beyond borders international (MBBI)	Trauma-Informed Peacebuilding and Development Assistance	NGO	USA	Not specified	Funders, multinational organizations, and practitioners in healthcare, education, human rights, conservation, mediation, and humanitarian aid	English proficiency	Flexible; agreed upon with partners	Flexible; agreed upon with partners	Case studies, vignettes, analysis of anecdotes, skills practice, small group discussion, reflection tasks, program design and simulation exercises
[50]	Minnesota Peacebuilding Leadership Institute	Introduction to Strategies for Trauma Awareness and Resilience for Cultural Competence, Racial Healing and Equity (Intro to STAR)	Academic institutions	USA	Not specified	Open to everyone, regardless of background or profession	English proficiency	2 h	Online	Not specified
[41]	Minnesota Peacebuilding Leadership Institute	Strategies for Trauma Awareness and Resilience (STAR) Training^a^	Academic institutions	USA	$995 - $1100; group discounts and scholarship support possible	Open to everyone, regardless of background or profession	English proficiency	38 h	In-person	Not specified
[42]	Mirovna Akademija & Peace Catalyst International	Trauma-sensitive Peacebuilding	NGO	Bosnia and Herzegovina	$600	People in caring professions (e.g. social workers, counselors, teachers, pastors, etc.), students and activists passionate about trauma healing and/or peacebuilding, and everyday people who are interested in getting involved in peacebuilding work in their communities	Application necessary, English proficiency	27 h (13 session x 2 h over 5 weeks + 1 h individual consultation) + self-paced learning	Online	Live online sessions, facilitated discussions, role playing, guest lectures, self-paced at-home learning, assignments at home, journaling, individual consultations
[34]	Northeast Asia Regional Peacebuilding Institute (NARPI)	NARPI Summer Peacebuilding Training	NGO	South Korea	$1300 incl. materials and lodging; scholarships available	NGO staff and interns, peace educators and activists, teachers and professors, students, government officials, military and police, community leaders, religious leaders, anyone interested in peacebuilding in North East Asia	Application necessary, some level of experience in peacebuilding required, English proficiency	5 days	In-person	Interactive sessions, case studies, participatory and experiential learning
[38]	ProPeace Academy for Conflict Transformation	Trauma-informed Peacebuilding	NGO	Germany	€405 - €450	Peace workers in international or domestic peace projects, as well as people working in active conflict zones	English proficiency	20 h (5 sessions x 4 h over 5 weeks) + self-paced learning	Online	Facilitated reflection and discussion in small and large groups, digital learning platform, case study analysis, project planning practice, self-paced learning, including reading assigned texts, listening to podcasts, watching videos, journaling, and inner mapping, alumni network
[35]	swisspeace	Mental well-being & peacebuilding	NGO	Switzerland	CHF1500	Peacebuilders, including persons working in the area of human rights, mediation, management and human resources of peacebuilding organizations, etc., anyone interested in the topic	University degree in a related discipline such as political science, law, economics or sociology, > 2 years professional experience, English proficiency	24 h (6 sessions x 4 h over 2 weeks)	Online	Expert input, Q&A, self reflection, group work, discussion, exercise, review
[51]	The Center for Justice and Peacebuilding (CJP), Eastern Mennonite University (EMU)	Strategies for Trauma Awareness and Resilience (STAR) - STAR1	Academic institutions	USA	$1054; scholarship available	Not specified	Application necessary, English proficiency	Online: 16 sessions x 2.5 h over 8 weeks In-person: 5- or 7-day formats	Online or in-person	Lectures, experiential and interactive activities, presentations, physical activity, creative expression, interaction with other participants
[36]	The Center for Justice and Peacebuilding (CJP), Eastern Mennonite University (EMU)	Strategies for Trauma Awareness and Resilience (STAR) - STAR2	Academic institutions	USA	$1054; scholarship available	Not specified	STAR 1 training, Application necessary, English proficiency	Online: 16 sessions x 2.5 h over 8 weeks In-person: 5- or 7-day formats	Online or in-person	Lectures, experiential and interactive activities, presentations, physical activity, creative expression, interaction with other participants
[39]	The Pocket Project	Applied Trauma-Informed Leadership Course	NGO	Germany	€945; Reductions for participants from crisis areas people of colour from the ‘Global South’ and indigenous leaders	Leaders in change movements, social enterprises, and NGOs	English proficiency	42 h over 4 months	Online	Live interactive online sessions, access to course materials, mentoring sessions, alumni network
**Non-facilitated training**:										
[53]	American Friends Service Committee (AFSC)	Trauma Healing and Psychosocial Support: A Training Manual for Development Practitioners and Case Care Workers	NGO	USA	No fee	Civil society and community- based organizations	Not specified	Self-paced	Document	Readings, case studies, discussions, practical exercise, reflection, activities
[48]	Corridors - Dialogue through Cooperation	Guidelines for Trauma Sensitivity in Peacebuilding and Community Work	NGO	Germany	No fee	Representatives of communities, peacebuilding organizations, and NGOs and civil-society organizations (CSOs), who work with communities after wars and armed conflicts	Not specified	Self-paced	Document	Readings
[45]	Mediators beyond borders international (MBBI)	Trauma-informed Practices	NGO	USA	$150	Not specified	English proficiency	1 h video content, 11 lessons	Web-based	Video content, readings
[49]	Mediators beyond borders international (MBBI)	Trauma-informed Peacebuilding in Ukraine	NGO	USA	Not specified	Not specified	Training available only upon request	1 h video content, 20 lessons	Web-based	Video content, readings
[46]	UNICEF North-Macedonia	Trauma-informed approach - A introductory handbook	International organization	North Macedonia	No fee	Judiciary, police, and social services	Not specified	Self-paced	Document	Readings, reflection
[54]	United Nation Institute for training and research (UNITAR)	Confronting trauma	International organization	Switzerland	No fee	People working in conflict and post conflict environments and the humanitarian sector, anyone experiencing stress in their daily and working lives	English proficiency	Self-paced	Web-based	Readings, stories, videos, activities, tests

^a^ A “STARS-Lite” version of the training (1 day) is also offered; however, it was not included in this review due to insufficient information on its content and structure

Training providers were located primarily in Western high-income countries, with ten (48%) in the United States, five (24%) in Germany, and three (14%) in Switzerland. One provider each was located in South Korea (5%), North Macedonia (5%), and Bosnia and Herzegovina (5%).

Most training programs targeted a broad audience regardless of professional background or prior experience. Only a few explicitly limited participation to practitioners with prior work experience in psychosocial support or peacebuilding [34–36]. Program duration ranged from short sessions (as brief as one hour) to multi-month online courses with weekly sessions or intensive in-person formats over several consecutive days. Fees ranged from $0-150 for non-facilitated programs, $450–1800 for facilitated online programs, and $995–1300 for in-person programs. Training methods included instructional methods (e.g., lectures, expert input), practice-oriented approaches (e.g., case studies, vignette), interactive and participatory methods (e.g., group discussions, role-plays, simulation exercises, reflective exercises), and self-paced learning (e.g., readings, videos, podcasts). Four training providers described providing follow-up support mechanisms such as WhatsApp groups, alumni networks, email-based mentoring, and access to online resources [37–40]. One training program reported learning outcomes based on a formal evaluation process [41].

### Key findings on recurring topics of trauma-informed peacebuilding training programs

The topics covered by the training programs overlapped considerably. Thematic analysis revealed seven recurring topics, including: (1) defining and conceptualizing trauma, (2) realizing the impact of trauma on peace and peacebuilding efforts, (3) developing self-care and resilience strategies for peace practitioners, (4) developing psychosocial support skills to address trauma and foster resilience in affected communities, (5) integrating trauma-informed practices into peacebuilding programs and organizations, (6) ethical considerations in trauma-informed peacebuilding, and (7) cultural competence and contextual awareness around trauma. These topics are described narratively in the following section. An overview of the topics is provided in Table 2.

**Table 2:** Recurring topics across training programs

Topics	No. (%) of trainings	References
Defining and conceptualizing trauma	21 (100%)	[34–54]
Realizing the impact of trauma on peace and peacebuilding efforts	19 (90%)	[34–51, 53]
Developing self-care and resilience strategies for peace practitioners	16 (76%)	[35, 36, 38–44, 46–48, 50–53]
Developing psychosocial support skills to address trauma and foster resilience in communities	15 (71%)	[34–38, 41, 44–51, 53]
Integrating trauma-informed practices into peacebuilding programs and organizations	14 (67%)	[35–44, 47, 52–54]
Ethical considerations in trauma-informed peacebuilding	11 (52%)	[37–43, 46, 48, 52, 53]
Cultural competence and contextual awareness around trauma	6 (29%)	[37–39, 42, 47, 48]

#### Defining and conceptualizing trauma

All training programs included a component on the definition and conceptualization of trauma. This included establishing a common understanding of trauma and distinguishing between different types of trauma. Most training programs incorporated a multidimensional and intersectional perspective, recognizing not only individual trauma but also the societal, structural and historical dimensions of trauma and their cumulative effects on individuals and communities [35, 38, 41–43]. Some training programs specifically mentioned the inclusion of information on the neurobiological basis of trauma and how trauma affects cognitive, emotional, and physiological functioning [44–46]. Furthermore, some programs provided more in-depth knowledge on how to recognize signs of stress and reactions caused by the experience of traumatic events [37, 38, 40–42]. This included information on symptoms of common trauma-related mental health problems and tools for assessing the psychological effects of trauma [37, 44].

#### Realizing the impact of trauma on peacebuilding

Nineteen training programs included information on how trauma can hinder peacebuilding by contributing to cycles of violence and aggression [34–36, 38–51]. In particular, this included training on the intersection between trauma and power dynamics, discussing how historical, structural and intergenerational trauma can undermine social cohesion and reconciliation efforts [40, 43, 50].

#### Developing self-care and resilience strategies for peace practitioners

Sixteen programs covered learning about the effects of primary and secondary trauma exposure on practitioners, highlighting its impact on individual well-being and team dynamics, including burnout, compassion fatigue, and stress [34–36, 38–44, 46–48, 50–53]. Training programs provided strategies and tools for self-care and resilience, such as self-awareness practices, reflection exercises, self-regulation techniques, and stress management training [39, 44, 53]. In addition, programs provided information on building supportive professional structures and practicing a duty of care for organizations in high-stress environments, focusing on support structures, fostering empathy and compassion in professional relationships, and developing skills to effectively support colleagues [38, 40, 43, 47].

#### Developing psychosocial support skills to address trauma and foster resilience in communities

Fourteen programs focused on building practical skills to provide psychosocial support to individuals and communities affected by trauma. Training included psychological first aid, basic systemic counseling, cognitive behavioral therapy, and arts-based approaches as methods for addressing trauma and promoting healing [34, 36–38, 41, 44, 51, 53]. In addition, programs included lessons on understanding and strengthening community resilience [36, 38, 41, 44–49, 51]. This included learning about strength-based approaches and processes for breaking cycles of violence to support individuals and communities in post-traumatic recovery and reconciliation [47, 48].

#### Integrating trauma-informed practices into program design and implementation

Fourteen programs provided training on embedding trauma-informed approaches into the design, delivery, and evaluation of peacebuilding projects [36–44, 47, 52–54]. This included training participants to integrate trauma sensitivity into conflict analysis and project planning [38, 40]. Moreover, training programs also provided guidance on applying trauma-sensitive principles within organizational structures and partner organizations as well as fostering networks and communities of practice of trauma-informed practitioners [35, 39, 43]. Additionally, some programs focused on training participants in advocating for expanded trauma treatment and mainstreaming trauma-informed approaches within peacebuilding efforts [47, 53, 54].

#### Ethical considerations and principles of trauma-informed practice

Eleven training programs described introducing participants to the basic principles of trauma-informed practice, including safety, trust, choice, collaboration, empowerment, and cultural sensitivity [37–44, 46, 48, 52, 53]. In addition, ethical considerations such as upholding dignity, recognizing common humanity, respecting diversity, and adhering to the principle of “do no harm” were addressed in some programs [38, 42]. Training programs also included reflections on histories of oppression, privilege and entitlement, as well as stereotypes and prejudice [38, 42].

#### Cultural competence and contextual awareness around trauma

Less than half of the training programs explicitly described adaptations of trauma-informed approaches to local contexts, including recognizing cultural variations in trauma experiences, expressions, and healing practices [37, 38, 42]. Some training programs specifically trained participants to foster cultural diversity as a team strength and promote trauma-informed team cultures that amplify marginalized voices and support inclusive healing [39].

## Discussion

To our knowledge, this is the first systematic mapping review examining training programs in the field of trauma-informed peacebuilding. A total of 21 training programs were identified, including 15 facilitated and six non-facilitated programs. Most training programs were provided by non-governmental organizations based in high-income Western countries. In addition to the limited global representation, structural challenges to the implementation and dissemination of trauma-informed peacebuilding training became apparent, including high participation fees and a lack of formal evaluation processes. Across available training programs, seven overlapping content components were identified. While several core components (e.g., defining trauma and understanding its impact) were consistently covered, other areas, particularly contextual adaptation and cultural awareness, were less commonly addressed. Overall, this review shows that training in trauma-informed peacebuilding remains fragmented, and suggests that structural features may limit engagement from practitioners in conflict-affected settings and low- and middle-income countries (LMICs). These findings underscore the urgent need for more inclusive, contextually grounded, and sustainably implemented training efforts that engage local practitioners and reflect local needs and resources.

There are significant geographical and structural imbalances in the current landscape of training programs for trauma-informed peacebuilding. The majority of identified programs is provided by non-governmental organizations based in high-income Western countries, with minimal representation of programs from conflict-affected settings or LMICs. High participation fees, language barriers, and selective eligibility criteria may further limit access for peacebuilding practitioners based in LMICs. These structural barriers can not only restrict access to training but also the ability of local actors to contribute to the design, adaptation, and dissemination of trauma-informed approaches. These challenges reflect long-standing critiques within the broader fields of MHPSS and peacebuilding [17, 55]. Scholars and practitioners in these fields have emphasized the limitations of top-down implementation models, Western-centric assumptions, and insufficient community ownership [17, 55]. Without deliberate efforts to shift these dynamics in the training of peacebuilding practitioners, there is a risk of reinforcing existing power asymmetries, further marginalizing those most directly involved in grassroots peacebuilding efforts and neglecting local knowledge and resources on the ground.

The reviewed training programs for trauma-informed peacebuilding consistently included a set of core components. These components include defining and conceptualizing trauma, understanding its individual and societal impact, developing strategies to prevent retraumatization and secondary trauma among clients and staff, and integrating trauma-informed practices into peacebuilding programs and organizations. These findings align with existing analyses of the core components of trauma-informed approaches in other sectors, such as mental health services or services for maltreated children [25, 26], as well as prevailing frameworks for trauma-informed practices [24]. Compared with trauma-informed approaches in other sectors, practitioner wellbeing appeared to receive particular emphasis across trauma-informed peacebuilding training programs. This likely reflects the challenging nature and specific pressures of peacebuilding work, particularly for practitioners who belong to conflict-affected communities or have been directly exposed to trauma [56]. Paying attention to practitioner well-being is not only an ethical imperative, but also has practical implications, as unaddressed distress can result in impaired decision-making, decrease productivity and reduce effectiveness of practitioners' peacebuilding efforts [21].

Notably, fewer than one third of training programs explicitly included modules focused on cultural competence, adaptations to specific conflict settings, or the integration of cultural practices and community resources. In the absence of such components, training content often appeared to rely on medicalized or Western-derived frameworks of mental health, which may not align with locally meaningful understandings of suffering and coping. This stands in contrast to calls from grassroots practitioners and local NGOs who emphasize that contextually grounded approaches are essential for addressing trauma and mental health in peacebuilding [15, 20]. As such, key dimensions of contextual responsiveness in peacebuilding settings include meaningful participatory engagement and trust-building with local communities, recognizing them as primary knowledge holders and ensuring that support programs align with local understandings, practices, and priorities in ways that foster ownership and agency [21]. Case studies from countries such as Uganda, Bosnia and Herzegovina, Haiti, Sri Lanka, Colombia, Syria, and South Sudan illustrate the diversity in how trauma and mental health are understood and addressed across settings [15, 20, 22, 23]. In peacebuilding interventions, responding to trauma may entail adapting language to avoid politicized terms, providing safe spaces to share war and social narratives, and addressing war-related trauma through traditional customs and psychosocial interventions [15, 20, 22]. This diversity underscores the importance of avoiding one-size-fits-all approaches and adapting trauma-informed work to local realities, needs, and resources. In line with this, research shows that trauma-informed and psychosocial interventions are more acceptable, effective, and best implemented when they are culturally adapted [57, 58]. The limited attention to contextual adaptation in current training programs for trauma-informed peacebuilding may therefore pose challenges for their relevance, local uptake of learnings, and sustainability.

Most training programs included modules aimed at building skills in psychosocial intervention or community-based mental health approaches. However, there remains considerable ambiguity around the intended scope of practice for peacebuilding practitioners. The training descriptions often remained unclear about whether participants are being prepared to use tools to recognize and refer individuals in distress or deliver low-intensity psychosocial interventions directly. This lack of role clarity reflects a broader debate regarding the integration of MHPSS and peacebuilding [21]. According to recent guidance, the aim of integration is not to turn peacebuilders into mental health professionals, or vice versa, but to foster referral pathways and intersectoral coordination mechanisms that enable a holistic response to conflict-related psychosocial challenges [21]. At the same time, it is important to acknowledge that in many conflict-affected regions, health systems are under-resourced and mental health professionals are scarce [59, 60]. In such contexts, task-sharing approaches and the delivery of structured, low-intensity psychosocial interventions by non-specialists, including peacebuilding practitioners, can be both appropriate and necessary [59, 60]. However, this requires explicit attention to supervision structures, ethical safeguards, referral mechanisms, and clearly defined boundaries of practice. It is important to recognize that peacebuilding and mental health professionals have different areas of expertise, which should complement rather than substitute for one another within support systems [21]. For example, peacebuilders may be trained to recognize signs of distress, provide psychological first aid when appropriate, and support community resilience [21]. Recognizing role boundaries for non-specialists, IASC Guidelines emphasize that people with severe mental health problems should be referred to specialized services for appropriate care [20, 61]. Training programs for trauma-informed peacebuilding should therefore be explicit about the competencies being taught, the expected boundaries of practice, and how practitioners can link with available services as part of an integrated support system.

Another key finding of this review is the lack of available information on training evaluation. It remains unclear whether training programs included formal evaluation mechanisms, as very few reported implementing any type of assessment. Notably, there is currently no peer-reviewed literature evaluating trauma-informed peacebuilding training programs, and only one of the reviewed programs reported a formal evaluation process with documented outcomes [41]. This mirrors concerns from other sectors, where trauma-related training has proliferated rapidly despite limited evaluation of its content quality or effectiveness [25]. The lack of systematic evaluation may not only limit the evidence base on what constitutes effective trauma-informed training, but potentially also hinders opportunities for learning, refinement, and scale-up. It also raises further important questions about the contextual relevance of existing programs. Without clear evaluation or feedback processes, it remains uncertain whether training content is aligned with the needs, lived experiences, and capacities of practitioners working across diverse humanitarian and peacebuilding settings. Future training initiatives would benefit from using evaluation frameworks that combine process evaluation (e.g. feasibility, acceptability, reach, and implementation barriers) with outcome-oriented assessment (e.g. changes in practitioner knowledge, skills, referral practices, or well-being). In peacebuilding contexts, such frameworks may also benefit from incorporating participatory and context-sensitive approaches that actively engage local practitioners and communities in defining meaningful outcomes and indicators of impact.

### Limitations

This review has several limitations. First, the search was restricted to English and German, which may have led to an underrepresentation of training programs available in other languages and may partially explain the high proportion of German and Swiss programs identified. Second, due to the scarcity of peer-reviewed literature on trauma-informed peacebuilding training, the review was limited to publicly available, non-bibliographic materials. While this approach allowed the review to analyze available training initiatives and strengthened practical relevance of the findings, the variability in the scope and quality of non-bibliographic material might have limited the ability to conduct a more robust assessment of training content and implementation. Findings from this review should be interpreted in light of the descriptive nature of the available data, with gaps and implications inferred from the publicly reported information rather than from formal evaluations.

### Implications

This review offers several critical implications for the design, implementation, and evaluation of trauma-informed peacebuilding training programs, with relevance for practitioners, policymakers, and researchers.

#### Design: towards inclusive and context-sensitive training

The identification of core components across programs provides a useful foundation for future training development. These core elements should be complemented by strong contextual and cultural adaptations to ensure relevance in diverse settings. As trauma can be experienced, understood, and addressed differently across sociocultural contexts, training programs should be designed or adapted in close collaboration with local actors and integrate local knowledge, practices, and resources. Community leadership and participatory design should be emphasized not only to ensure contextual relevance, but also to address existing power imbalances in global training design and opportunities. To improve inclusivity, existing programs should also reflect and reduce structural barriers to participation, such as language, cost, and eligibility restrictions.

#### Implementation: promoting scalability and sustainability

Trauma-informed peacebuilding training programs should incorporate implementation strategies that promote sustainability and scalability. This may include the development of decentralized training models, such as train-the-trainer models, and the creation of open-access and adaptable materials, such as modular toolkits, facilitation guides, and translated manuals. Structured opportunities for follow-up, such as mentoring, supervision, or communities of practice, should be included to reinforce continuous learning and application over time. The use of digital tools can support these efforts. Flexibility to tailor to context-specific needs can be further enhanced through pre-training needs assessments, open sessions to incorporate emerging local themes, and built-in mechanisms for feedback and iterative adaptation.

#### Evaluation: strengthening learning and accountability

The absence of robust evaluation practices represents a critical gap in current training efforts. Future programs should conduct and report systematic documentation of training design, implementation processes, and training outcomes. Rigorous evaluation is essential to assess effectiveness, ensure accountability, and support continuous improvement. Research can help refine training content, improve delivery methods, and contribute to a stronger evidence base to guide future program development and policy decisions in peacebuilding.

## Conclusion

Given the profound impact of trauma in (post-)conflict settings, training in trauma-informed peacebuilding plays a critical role in equipping practitioners and organizations to support individual and collective healing, foster social cohesion, and break cycles of violence. While there appears to be broad consensus on some core components, such as understanding trauma and its psychosocial consequences, substantial gaps in the landscape of available trauma-informed peacebuilding training programs remain. Notably, a lack of global representation, limited contextual adaptation, insufficient engagement of local practitioners, and a lack of evaluation potentially undermine the relevance, accessibility, and sustainability of current training efforts. Future training initiatives must adopt inclusive, context-sensitive approaches that integrate local knowledge, promote practical application, and embed mechanisms for ongoing support and evaluation to effectively address trauma and meaningfully contribute to long-term peacebuilding.

## Supplementary Information

Supplementary Material 1.

## Data Availability

No datasets were generated or analysed during the current study.
