# Supplementary material for: Trauma-informed peacebuilding: a systematic mapping review of training programs
Source: Confl Health. 2026 Feb 19;20:18. doi: 10.1186/s13031-026-00773-6 (PMC12930582; doi:10.1186/s13031-026-00773-6)
Supplement: Supplementary file 1 — Supplementary Material 1. [file 13031_2026_773_MOESM1_ESM.docx]

**Supplement**

**Supplement S1. Query for initial search of bibliographic material**

| **Database** | **Search query** | **# results** | **# results matching eligibility criteria** | **Date of search** |
| --- | --- | --- | --- | --- |
| Web of Science | (trauma OR “trauma-informed” OR “trauma-healing” OR “trauma-sensitive” OR “trauma-aware*” OR traumatization OR traumatized) AND (peacebuilding OR peacekeeping OR "conflict transformation" OR “peace practitioner*” OR peacebuilders) AND (training OR course OR workshop OR “capacity building” OR education) (All Fields) | 41 | 0 | 25.11.24 |
| Scopus | TITLE-ABS-KEY ((trauma OR "trauma-informed" OR "trauma-healing" OR "trauma-sensitive" OR "trauma-aware*" OR traumatization OR traumatized ) AND ( peacebuilding OR peacekeeping OR "conflict transformation" OR "peace practitioner*" OR peacebuilders ) AND ( training OR course OR workshop OR "capacity building" OR education)) | 47 | 0 | 25.11.24 |
| PubMed | ((trauma OR "trauma-informed" OR "trauma-healing" OR "trauma-sensitive" OR "trauma-aware*" OR traumatization OR traumatized ) AND ( peacebuilding OR peacekeeping OR "conflict transformation" OR "peace practitioner*" OR peacebuilders ) AND ( training OR course OR workshop OR "capacity building" OR education)) | 71 | 0 | 25.11.24 |

**Supplement S2. PRISMA-ScR Checklist**

**PRISMA 2020 Main Checklist**

From: Page MJ, McKenzie JE, Bossuyt PM, Boutron I, Hoffmann TC, Mulrow CD, et al. The PRISMA 2020 statement: an updated guideline for reporting systematic reviews. MetaArXiv. 2020, September 14. DOI: 10.31222/osf.io/v7gm2. For more information, visit: [www.prisma-statement.org](file:///Users/larsdumke/Downloads/www.prisma-statement.org)

| **Topic** | **No.** | **Item** | **Location where item is reported** |
| --- | --- | --- | --- |
| **TITLE** |  |  |  |
| **Title** | 1 | Identify the report as a systematic review. | Page 1 |
| **ABSTRACT** |  |  |  |
| **Abstract** | 2 | See the PRISMA 2020 for Abstracts checklist | Page 2 |
| **INTRODUCTION** |  |  |  |
| **Rationale** | 3 | Describe the rationale for the review in the context of existing knowledge. | Page 3-4 |
| **Objectives** | 4 | Provide an explicit statement of the objective(s) or question(s) the review addresses. | Page 4 |
| **METHODS** |  |  |  |
| **Eligibility criteria** | 5 | Specify the inclusion and exclusion criteria for the review and how studies were grouped for the syntheses. | Page 5 |
| **Information sources** | 6 | Specify all databases, registers, websites, organisations, reference lists and other sources searched or consulted to identify studies. Specify the date when each source was last searched or consulted. | Page 5 |
| **Search strategy** | 7 | Present the full search strategies for all databases, registers and websites, including any filters and limits used. | Page 6, Page 33, Supplement S3 |
| **Selection process** | 8 | Specify the methods used to decide whether a study met the inclusion criteria of the review, including how many reviewers screened each record and each report retrieved, whether they worked independently, and if applicable, details of automation tools used in the process. | Page 5-6 |
| **Data collection process** | 9 | Specify the methods used to collect data from reports, including how many reviewers collected data from each report, whether they worked independently, any processes for obtaining or confirming data from study investigators, and if applicable, details of automation tools used in the process. | Page 5-6 |
| **Data items** | 10a | List and define all outcomes for which data were sought. Specify whether all results that were compatible with each outcome domain in each study were sought (e.g. for all measures, time points, analyses), and if not, the methods used to decide which results to collect. | Page 6 |
|  | 10b | List and define all other variables for which data were sought (e.g. participant and intervention characteristics, funding sources). Describe any assumptions made about any missing or unclear information. | Page 6 |
| **Study risk of bias assessment** | 11 | Specify the methods used to assess risk of bias in the included studies, including details of the tool(s) used, how many reviewers assessed each study and whether they worked independently, and if applicable, details of automation tools used in the process. | Page 5, Supplement S4 |
| **Effect measures** | 12 | Specify for each outcome the effect measure(s) (e.g. risk ratio, mean difference) used in the synthesis or presentation of results. | N/A |
| **Synthesis methods** | 13a | Describe the processes used to decide which studies were eligible for each synthesis (e.g. tabulating the study intervention characteristics and comparing against the planned groups for each synthesis (item 5)). | Page 6 |
|  | 13b | Describe any methods required to prepare the data for presentation or synthesis, such as handling of missing summary statistics, or data conversions. | N/A |
|  | 13c | Describe any methods used to tabulate or visually display results of individual studies and syntheses. | Page 6 |
|  | 13d | Describe any methods used to synthesize results and provide a rationale for the choice(s). If meta-analysis was performed, describe the model(s), method(s) to identify the presence and extent of statistical heterogeneity, and software package(s) used. | N/A |
|  | 13e | Describe any methods used to explore possible causes of heterogeneity among study results (e.g. subgroup analysis, meta-regression). | N/A |
|  | 13f | Describe any sensitivity analyses conducted to assess robustness of the synthesized results. | N/A |
| **Reporting bias assessment** | 14 | Describe any methods used to assess risk of bias due to missing results in a synthesis (arising from reporting biases). | N/A |
| **Certainty assessment** | 15 | Describe any methods used to assess certainty (or confidence) in the body of evidence for an outcome. | N/A |
| **RESULTS** |  |  |  |
| **Study selection** | 16a | Describe the results of the search and selection process, from the number of records identified in the search to the number of studies included in the review, ideally using a flow diagram. | Page 6 |
|  | 16b | Cite studies that might appear to meet the inclusion criteria, but which were excluded, and explain why they were excluded. | N/A |
| **Study characteristics** | 17 | Cite each included study and present its characteristics. | Page 20-22  Table 1 |
| **Risk of bias in studies** | 18 | Present assessments of risk of bias for each included study. | Page 35 Supplement S4 |
| **Results of individual studies** | 19 | For all outcomes, present, for each study: (a) summary statistics for each group (where appropriate) and (b) an effect estimate and its precision (e.g. confidence/credible interval), ideally using structured tables or plots. | N/A |
| **Results of syntheses** | 20a | For each synthesis, briefly summarise the characteristics and risk of bias among contributing studies. | Page 7-9 |
|  | 20b | Present results of all statistical syntheses conducted. If meta-analysis was done, present for each the summary estimate and its precision (e.g. confidence/credible interval) and measures of statistical heterogeneity. If comparing groups, describe the direction of the effect. | N/A |
|  | 20c | Present results of all investigations of possible causes of heterogeneity among study results. | N/A |
|  | 20d | Present results of all sensitivity analyses conducted to assess the robustness of the synthesized results. | N/A |
| **Reporting biases** | 21 | Present assessments of risk of bias due to missing results (arising from reporting biases) for each synthesis assessed. | N/A |
| **Certainty of evidence** | 22 | Present assessments of certainty (or confidence) in the body of evidence for each outcome assessed. | N/A |
| **DISCUSSION** |  |  |  |
| **Discussion** | 23a | Provide a general interpretation of the results in the context of other evidence. | Page 10 |
|  | 23b | Discuss any limitations of the evidence included in the review. | Page 13 |
|  | 23c | Discuss any limitations of the review processes used. | Page 13-14 |
|  | 23d | Discuss implications of the results for practice, policy, and future research. | Page 14-15 |
| **OTHER INFORMATION** |  |  |  |
| **Registration and protocol** | 24a | Provide registration information for the review, including register name and registration number, or state that the review was not registered. | Page 4 |
|  | 24b | Indicate where the review protocol can be accessed, or state that a protocol was not prepared. | N/A |
|  | 24c | Describe and explain any amendments to information provided at registration or in the protocol. | N/A |
| **Support** | 25 | Describe sources of financial or non-financial support for the review, and the role of the funders or sponsors in the review. | Page 1 |
| **Competing interests** | 26 | Declare any competing interests of review authors. | Page 1 |
| **Availability of data, code and other materials** | 27 | Report which of the following are publicly available and where they can be found: template data collection forms; data extracted from included studies; data used for all analyses; analytic code; any other materials used in the review. | Pages 16-19 |

**PRIMSA Abstract Checklist**

| **Topic** | **No.** | **Item** | **Reported?** |
| --- | --- | --- | --- |
| **TITLE** |  |  |  |
| **Title** | 1 | Identify the report as a systematic review. | Yes |
| **BACKGROUND** |  |  |  |
| **Objectives** | 2 | Provide an explicit statement of the main objective(s) or question(s) the review addresses. | Yes |
| **METHODS** |  |  |  |
| **Eligibility criteria** | 3 | Specify the inclusion and exclusion criteria for the review. | Yes |
| **Information sources** | 4 | Specify the information sources (e.g. databases, registers) used to identify studies and the date when each was last searched. | Yes |
| **Risk of bias** | 5 | Specify the methods used to assess risk of bias in the included studies. | No |
| **Synthesis of results** | 6 | Specify the methods used to present and synthesize results. | Yes |
| **RESULTS** |  |  |  |
| **Included studies** | 7 | Give the total number of included studies and participants and summarise relevant characteristics of studies. | Yes |
| **Synthesis of results** | 8 | Present results for main outcomes, preferably indicating the number of included studies and participants for each. If meta-analysis was done, report the summary estimate and confidence/credible interval. If comparing groups, indicate the direction of the effect (i.e. which group is favoured). | Yes |
| **DISCUSSION** |  |  |  |
| **Limitations of evidence** | 9 | Provide a brief summary of the limitations of the evidence included in the review (e.g. study risk of bias, inconsistency and imprecision). | Yes |
| **Interpretation** | 10 | Provide a general interpretation of the results and important implications. | Yes |
| **OTHER** |  |  |  |
| **Funding** | 11 | Specify the primary source of funding for the review. | Yes |
| **Registration** | 12 | Provide the register name and registration number. | N/A |

**Supplement S3. Systematic search strategy**

| **Search term** | **# results screened on Google** | **# results screened on Metacrawler** | **Date of search** |
| --- | --- | --- | --- |
| Trauma peacebuilding training | 100 | 100 | 08.12.24 |
| “Trauma-sensitive” peacebuilding training | 100 | 100 | 08.12.24 |
| “Trauma-informed” peacebuilding training | 100 | 100 | 08.12.24 |
| “Trauma-aware*” peacebuilding training | 100 | 100 | 08.12.24 |
| Trauma peacebuilding course | 100 | 100 | 08.12.24 |
| “Trauma-sensitive” peacebuilding course | 100 | 100 | 08.12.24 |
| “Trauma-informed” peacebuilding course | 100 | 100 | 08.12.24 |
| “Trauma-aware*” peacebuilding course | 100 | 100 | 08.12.24 |
| Trauma peacebuilding practice | 100 | 100 | 08.12.24 |
| “Trauma-sensitive” peacebuilding practice | 100 | 100 | 08.12.24 |
| “Trauma-informed” peacebuilding practice | 100 | 100 | 08.12.24 |
| “Trauma-aware*” peacebuilding practice | 100 | 100 | 08.12.24 |

**Supplement S4. Critical Appraisal based on AACODS (Authority, Accuracy, Coverage, Objectivity, Date, Significance) Checklist**

| **Ref.** | **Name of Training Provider** | **Title of Training Program** | **AACODS score** ^a^ |
| --- | --- | --- | --- |
|  | Facilitated training: |  |  |
| [1] | Alliance for Middle East Peace (ALLMEP) | Trauma in Peacebuilding | 4 |
| [2] | Hartford International University for Religion and Peace | Peacebuilding Skills: Dialogue, Trauma & Restorative Justice | 5 |
| [3] | International Association for Human Values (IAHV) | Towards Integrated Peacebuilding | 5 |
| [4] | Kurve Wustrov | Stress and Trauma Sensitivity in Peacebuilding | 5 |
| [5] | Center for International Peace Operations (ZIF) | Trauma-Sensitivity in Peace Operations | 6 |
| [6] | Mediators beyond borders international (MBBI) | Trauma-Informed Peacebuilding and Development Assistance | 4 |
| [7] | Minnesota Peacebuilding Leadership Institute | Introduction to Strategies for Trauma Awareness and Resilience for Cultural Competence, Racial Healing and Equity (Intro to STAR) | 4 |
| [8] | Minnesota Peacebuilding Leadership Institute | Strategies for Trauma Awareness and Resilience (STAR) Training^a^ | 4 |
| [9] | Mirovna Akademija & Peace Catalyst International | Trauma-sensitive Peacebuilding | 6 |
| [10] | Northeast Asia Regional Peacebuilding Institute (NARPI) | [NARPI Summer Peacebuilding Training](http://narpi.net/?page_id=7#program-01) | 5 |
| [11] | ProPeace Academy for Conflict Transformation | Trauma-informed Peacebuilding | 6 |
| [12] | swisspeace | Mental well-being & peacebuilding | 6 |
| [13] | The Center for Justice and Peacebuilding (CJP), Eastern Mennonite University (EMU) | Strategies for Trauma Awareness and Resilience (STAR) - STAR1 | 6 |
| [14] | The Center for Justice and Peacebuilding (CJP), Eastern Mennonite University (EMU) | Strategies for Trauma Awareness and Resilience (STAR) - STAR2 | 6 |
| [15] | The Pocket Project | Applied Trauma-Informed Leadership Course | 5 |
|  | Non-facilitated training: |  |  |
| [16] | American Friends Service Committee (AFSC) | Trauma Healing and Psychosocial Support: A Training Manual for Development Practitioners and Case Care Workers | 4 |
| [17] | Corridors - Dialogue through Cooperation | Guidelines for Trauma Sensitivity in Peacebuilding and Community Work | 4 |
| [18] | Mediators beyond borders international (MBBI) | Trauma-informed Practices | 3 |
| [19] | Mediators beyond borders international (MBBI) | Trauma-informed Peacebuilding in Ukraine | 3 |
| [20] | UNICEF North-Macedonia | Trauma-informed approach - A introductory handbook | 4 |
| [21] | United Nation Institute for training and research (UNITAR) | Confronting trauma | 5 |

Notes. ^a^ Scores range from 0-6.

**References**

1. ALLMEP. Trauma in Peacebuilding. Alliance for Middle East Peace. https://www.allmep.org/trauma-in-peacebuilding/. Accessed 11 Mar 2025.

2. Hartford International University for Religion and Peace. Peacebuilding Skills: Dialogue, Trauma & Restorative Justice. https://www.hartfordinternational.edu/courses/peacebuilding-skills-dialogue-trauma-restorative-justice. Accessed 11 Mar 2025.

3. IAHV Peacebuilding. Towards Integrated Peacebuilding: Psychosocial Wellbeing, Skills and Knowledge for Increased Peacebuilding Impact. https://iahv-peace.org/. https://iahv-peace.org/services/professional-training-towards-integrated-peacebuilding-psychosocial-skills-and-professional-care-for-peacebuilders-in-the-field/. Accessed 11 Mar 2025.

4. KURVE Wustrow. Stress and Trauma Sensitivity in Peacebuilding. 2025. https://www.kurvewustrow.org/en/termin/stress-and-trauma-sensitivity-peacebuilding. Accessed 11 Mar 2025.

5. ZIF. Trauma-Sensitivity in Peace Operations. https://www.zif-berlin.org/en/trauma-sensitivity-peace-operations-remote-eng. Accessed 11 Mar 2025.

6. MBBI. Trauma-Informed Peacebuilding & Development Assistance. Mediators Beyond Borders International. https://mediatorsbeyondborders.org/what-we-do/trauma-informed-peacebuilding/. Accessed 11 Mar 2025.

7. Minnesota Peacebuilding Leadership Institute. Introduction to Strategies for Trauma Awareness and Resilience for Cultural Competence, Racial Healing and Equity. Minnesota Peacebuilding Leadership Institute. https://www.mnpeace.org/intro-to-star-2-hr.html. Accessed 11 Mar 2025.

8. Minnesota Peacebuilding Leadership Institute. Strategies for Trauma Awareness and Resilience. Minnesota Peacebuilding Leadership Institute. https://www.mnpeace.org/star-training-5-days.html. Accessed 11 Mar 2025.

9. Mirovna Akademija. Trauma-Sensitive Peacebuilding Course. https://www.mirovna-akademija.org/rma/en/education/trauma-sensitive-peacebuilding-program-introduction-course-description. Accessed 11 Mar 2025.

10. NARPI. NARPI Peacebuilding Training. http://narpi.net/?page_id=7. Accessed 11 Mar 2025.

11. Pro Peace Academy. Trauma-informed Peacebuilding. 2025. https://akademie.propeace.de/en/course/trauma-informed-peacebuilding-0. Accessed 11 Mar 2025.

12. swisspeace. Mental Wellbeing & Peacebuilding. https://www.swisspeace.ch/courses/short-courses/mental-wellbeing-and-peacebuilding. Accessed 29 May 2025.

13. The Center for Justice and Peacebuilding. STAR Level 1 Training. https://emu.edu/cjp/star/training-options#starone. Accessed 11 Mar 2025.

14. The Center for Justice and Peacebuilding. STAR Level 2 Training. https://emu.edu/cjp/star/training-options#startwo. Accessed 11 Mar 2025.

15. The Pocket Project. Applied Trauma-Informed Leadership Course. The Pocket Project. https://pocketproject.org/applied-trauma-informed-leadership-course/. Accessed 11 Mar 2025.

16. American Friends Service Committee. Trauma Healing and Psychosocial Support: A Training Manual for Development Practitioners and Case Care Workers.

17. Corridors. Trauma Informed Peacebuilding. Corridors. http://www.opencorridors.de/our-work-1/trauma-informed-peacebuilding/. Accessed 11 Mar 2025.

18. MBBI. Trauma-Informed Peacebuilding. Mediators Beyond Borders International. https://mbbi.thinkific.com/courses/trauma-informed-practice. Accessed 11 Mar 2025.

19. MBBI. Травма-інформована розбудова миру в Україні. Mediators Beyond Borders International. https://mbbi.thinkific.com/courses/ukraine-trauma-informed-peacebuilding. Accessed 11 Mar 2025.

20. UNICEF North Macedonia. Trauma informed approach. 2022. https://www.unicef.org/northmacedonia/reports/trauma-informed-approach. Accessed 11 Mar 2025.

21. UNITAR. Confronting Trauma [PTP.2024.12E]. https://unitar.org/courses/confronting-trauma-ptp202412e-11202. Accessed 11 Mar 2025.
